# Aqueous-soluble bipyridine cobalt(ii/iii) complexes act as direct redox mediators in photosystem I-based biophotovoltaic devices

**DOI:** 10.1039/d0ra10221k

**Published:** 2021-03-11

**Authors:** Alexandra H. Teodor, Eu-Jee Ooi, Jackeline Medina, Miguel Alarcon, Michael D. Vaughn, Barry D. Bruce, Jesse J. Bergkamp

**Affiliations:** Graduate School of Genome Science and Technology, University of Tennessee at Knoxville and Oak Ridge National Laboratory USA bbruce@utk.edu; Department of Chemistry and Biochemistry, California State University Bakersfield USA jbergkamp@csub.edu; SpectroLogix Knoxville TN USA; Department of Biochemistry & Cellular and Molecular Biology, University of Tennessee at Knoxville USA; Department of Chemical and Biomolecular Engineering, University of Tennessee at Knoxville USA

## Abstract

Sustainable energy production is critical for meeting growing worldwide energy demands. Due to its stability and reduction potential, photosystem I (PSI) is attractive as the photosensitizer in biophotovoltaic devices. Herein, we characterize aqueous and organic solvent soluble synthetic bipyridine-based cobalt complexes as redox mediators for PSI-based biophotovoltaics applications. Cobalt-based complexes are not destructive to protein and have appropriate midpoint potentials for electron donation to PSI. We report on PSI stability in organic solvents commonly used in biophotovoltaics. We also show the effects of a mixed organic solvent phase on PSI reduction kinetics, slowing reduction rates approximately 8–38 fold as compared to fully aqueous systems, with implications for dye regeneration rates in PSI-based biophotovoltaics. Further, we show evidence of direct electron transfer from cobalt complexes to PSI. Finally, we report on photocurrent generation from Co mediator-PSI biophotovoltaic devices. Taken together, we discuss the development of novel Co complexes and our ability to fine-tune their characteristics *via* functional groups and counteranion choice to drive interaction with a biological electron acceptor on multiple levels from redox midpoints, spectral overlap, and solvent requirements, among others. This work suggests that fine-tuning of redox active species for interaction with a biological partner is possible for the creation and improvement of low cost, carbon-neutral energy production in the future.

## Abstract 2

Sustainable energy production is critical for meeting growing worldwide energy demands.

## Graphical abstract

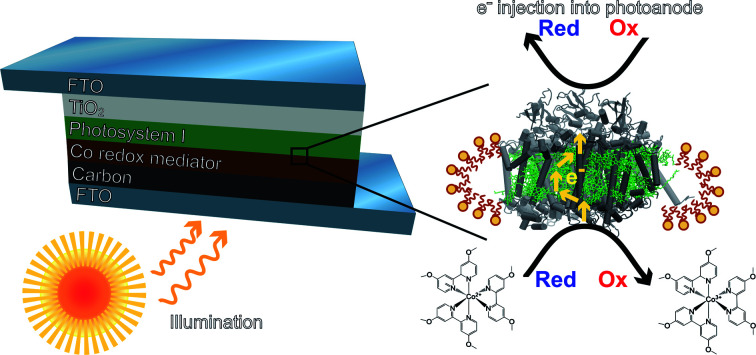

## Introduction

One of the greatest problems facing the planet today is the development of sustainable energy sources. The world's energy demand is predicted to increase nearly 40% by 2040, and supplies of fossil fuels are by their nature limited.^1^ As such, there is a need for carbon-neutral, sustainable, and inexpensive methods of energy production to help meet this growing demand. The development of photovoltaic technologies utilizing abundant, sustainable resources and manufacturing methods is of great interest. One such type of photovoltaic device is the dye-sensitized solar cell (DSSC), using relatively abundant titanium dioxide as semiconductor.^2^

**Fig. 1:**
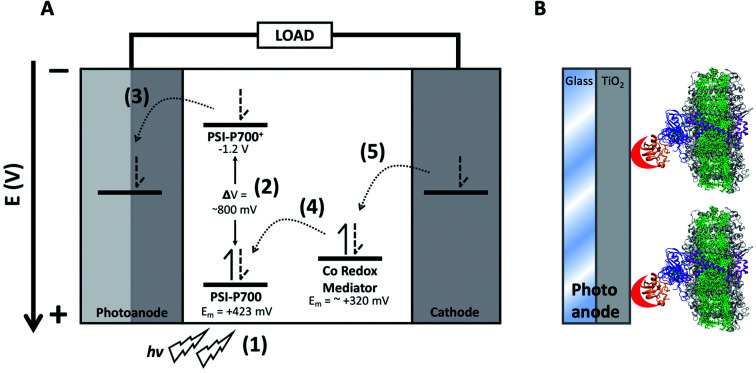
Structure of a PSI-based DSSC. Diagram of electron transfer in a PSI-based DSSC. (A) Numbers in parenthesis denote electron transfer steps: (1) PSI absorbs a photon, (2) which then promotes the special chlorophyll pair PSI-P700 to an excited state. (3) Charge separation occurs and the electron is injected into the anode (composed of transparent conductive glass and TiO_2_) occurs. (4) The redox mediator reduces the oxidized PSI-P700^+^ back to the ground state. (5) The mediators then undergo reversible reduction at the cathode, completing the circuit. (B) Schematic showing the design of a PSI-based photoanode. Incorporation of PSI onto the TiO_2_ photoanode surface of a DSSC, the TiO_2_ is shown in gray. PSI subunit PsaF which promotes the receiving of electrons from redox mediators in algae and higher plants is shown in purple, and subunits PsaC, D, and E are shown in blue and are chemically crosslinked to ferredoxin, shown in orange. A modified metal-binding peptide on the end of ferredoxin is shown in red that binds onto the TiO_2_ surface on the anode, allowing for directional, constructive electron transfer to occur.

Though DSSCs have only achieved an external quantum efficiency (EQE) approximately one-third that of current single-junction PV cells, they have some unique advantages^3^ that include the ability to be constructed out of readily available materials and their amenability for modification and optimization. All DSSCs are comprised of five primary components: a photoexcitable dye, a counter electrode, a transparent electrode enabling photoexcitation of the dye, a conductive semiconductor, and one or more redox mediators in an electrolyte solution.^3^ Although many plant pigments have been shown to function in DSSCs,^4,5^ the field of applied photosynthesis offers the potential of using the highly abundant and stable photosynthetic reaction center photosystem I (PSI) as the photosensitive dye in bio-hybrid DSSCs.^6,7^ Compared to simple plant pigments, PSI is a reaction center that utilizes pigments as a light harvesting antenna coordinated by a protein scaffold, yet also coordinates charge separation *via* a specially oriented pair of pigments. A diagram demonstrating incorporation of PSI into a DSSC can be seen in Fig. 1, where the photosensitive dye PSI is bound onto a transparent conductive glass electrode with a TiO_2_ semiconductor layer and oriented for forward electron injection into the anode using a metal-binding peptide crosslinked onto the native PSI electron acceptor protein, ferredoxin.

In photosynthetic reaction centers, chlorophyll pigments absorb photons and are excited to a higher energy state, and pass exciton energy to a pair of closely oriented chlorophyll known as a “special pair” where charge separation occurs with an internal quantum efficiency approaching 100%.^8,9^ In PSI, this special pair is referred to as P700.^8^ A large amount of applied photosynthesis research has focused on PSI from the thermophilic cyanobacterium *Thermosynechococcus elongatus* BP-1 (*T. elongatus*). The selection of this organism is in part due its relatively high thermostability, early availability of a sequenced and annotated genome, a robust and straight forward isolation procedure, the ability to be genetically transformed^10–12^ and most importantly the first availability of a high-resolution 3D PSI structure.^13^ Although all PSI complexes can generate a powerful reducing potential of approximately −1.2 V,^10^ prior work has shown that PSI from *T. elongatus* can remain photoactive after isolation for well over 90 days.^10^

An abbreviated summary of photoelectrochemical electrodes and devices made utilizing PSI and the various properties of these are listed in Table 1 below, with focus on the redox mediators and solvents used for each. Wiring of PSI to the electrode surface is performed *via* a number of methods including drop-casting, oriented binding of PSI/PSI electron acceptors *via* a metal-binding peptide or molecular wire, self-assembly of mono- or multi-layers, or chemical crosslinking onto functionalized electrode surfaces. The redox mediator described herein and for the rest of this manuscript refers solely to the component in the electrolyte used for reducing PSI from its photo-oxidized state, closing the circuit between half-reactions.

**Table 1:** Comparison of PSI-based electrode and photovoltaic device current densities. Comparisons between a representative sample of PSI-based half-cells and full photoelectrochemical devices. Current densities were normalized to intensity of the excitation light

Electrode or full device	PSI – electrode surface	Redox mediator	Solvent	Current density (μA cm^−2^ mW^−1^)	Ref.
Device	TiO_2_/ZnO-PsaD/E-PSI	Z813 Co(ii/iii)	60% ethylene carbonate, 40% acetonitrile	4469.1	30
Electrode	Au	Os(bpy)Cl_2_ embedded in redox polymers P1, P2, P3	Aqueous	16.1	31
Electrode	Au on ITO TiO_2_	Os(bpy)Cl_2_ embedded in Nafion	Aqueous	1.07	32
Electrode	Au NP/SAM/MPS/PSI	Ascorbate/DCPIP	Aqueous	0.48	33
Electrode	NQC15EV on Au electrode	Ascorbate/DCPIP	Aqueous	0.125	34
Device	Au on Si support	Ascorbate/DCIP	Aqueous	0.032	35
Electrode	NQC15S-Au NP on Au	Ascorbate/DCPIP	Aqueous	0.007	36

However, current designs still function well below the internal quantum efficiency shown for PSI and photosynthetic charge separation in general,^9,14^ suggesting room for device improvement. There are many different approaches being studied for the improvement of PSI-based biohybrid devices, including improving the unidirectionality of deposited PSI on electrodes,^15,16^ improvement of the optical cross-section of PSI to increase light harvesting capabilities,^17,18^ and interest in creation of solid-state devices^19–21^ A key area of improvement is in PSI reduction rates to help increase dye regeneration rates. Reduction of *T. elongatus* PSI by its native biological redox mediator, the one-electron shuttle protein cytochrome *c*_6_, has been shown to be slow relative to the same process in other cyanobacteria and algae, and approximately 1000 times slower when compared to higher plants.^22–25^ The long-lived charge separated state caused by this slow reduction step allows for charge recombination to take place, wasting photonic energy. Furthermore, the modification of cytochrome *c*_6_ to enhance electron transfer rates to PSI has proven to be less straightforward than expected; an exact reaction mechanism for PSI-cytochrome *c*_6_ interaction and electron transfer remains elusive, hindering point mutations and other modifications to enhance electron transfer rates.^22–29^ Switching from protein-based mediators to organometallic complexes will allow enhanced stability and more fine control of functional groups, as well as bypassing any protein docking steps that may be rate limiting. Herein, we present characterization of multiple cobalt-based redox mediators based on bipyridine ligands and their ability to reduce PSI in solution, along with optimization for complementing the needs of these synthetic redox mediators with the biological PSI to retain and enhance activity. We show results suggesting decreased structural stability of PSI in the organic solvent acetonitrile, with leaching of approximately 20% of the chlorophyll out of the PSI complex. We also show an approximately 8-fold increase in PSI reduction rates when moving from a buffer system utilizing organic solvents to a fully aqueous buffer system when the small molecule 2,6-dichlorophenolindophenol (DCPIP) is used, and a nearly 38-fold increase in PSI reduction rates for one of our cobalt complexes when moving from organic solvent to fully aqueous buffer systems. Finally, we also present data on PSI-specific photocurrent generation in photovoltaic devices utilizing our aqueous-soluble mediator and comparison to the ternary solvent-based electrolyte system reported on previously in the literature.

## Materials and methods

### Isolation of PSI

PSI was isolated and purified from *T. e.* as previously reported.^10^ Briefly, frozen *T. elongatus* frozen cells were resuspended in wash buffer and Dounce homogenized. Lysozyme was added and the suspension was incubated to allow for cell wall degradation. The suspension was pelleted and washed with fresh wash buffer before being passed twice through a French Press. The lysate was centrifuged, and the pelleted membrane fragments were washed again. *N*-dodecyl β-d-maltoside (β-DDM) was added to the resuspended pellet, which was then incubated. The insoluble material was removed *via* centrifugation and the solubilized material from the membrane pellet was then separated using sucrose density gradient ultracentrifugation, after which the lowest band containing trimeric PSI was harvested. Harvested PSI was then purified using HPLC before aliquoting and storage.

### Cobalt redox mediator complex synthesis

**Scheme 1:**
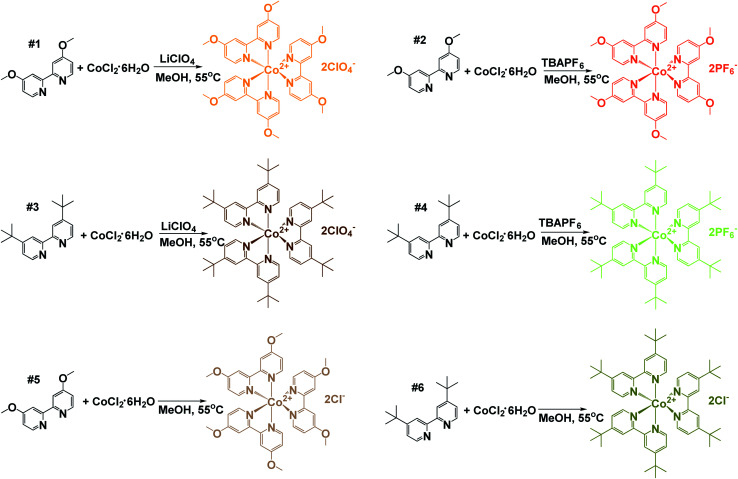
Synthetic schemes for the preparation of tris(4,4′-di-*tert*-butyl-2,2′-bipyridine)cobalt(ii) and tris(4,4′-di-methoxy-2,2′-bipyridine)cobalt(ii) complexes. Three equivalents of each indicated bipyridine ligand was reacted with one equivalent of cobalt chloride hexahydrate in methanol at 55 °C for approximately 3 h. The resulting solution containing the Co(bpy-R_2_)_3_Cl_2_ complex was split into two round-bottom flasks and the corresponding counteranions were introduced. Ten equivalents of tetrabutylammoniumhexafluorophosphate (TBAPF_6_) or lithium perchlorate (LiClO_4_) were added to the designated flask. The resulting solid was isolated *via* vacuum filtration and washed with cold methanol followed by cold ethyl ether. To obtain the chloride salt, the original reaction was evaporated under reduced pressure and resulting solid washed with cold ethyl ether.

Two bipyridine cobalt complexes bearing methoxy groups and two bipyridine cobalt complexes bearing *tert*-butyl groups were synthesized, using perchlorate, hexafluorophosphate, or chloride counterions. Synthetic schemes to form the six cobalt redox mediator complexes are shown in Scheme 1. Complexes 1 and 2 were formed by using 3 molar equivalents of 4,4′-dimethoxy-2,2′-bipyridine which was reacted with 1 molar equivalent of cobalt chloride hexahydrate in methanol for 3–4 hours at 55 °C. The solution was divided into two separate batches, and two subsequent reactions were performed for counterion addition. Complexes 3 and 4 utilized 4,4′-di-*tert*-butyl-2,2′-bipyridine as coordinating ligands. To obtain complexes 5 and 6, 3 molar equivalents of ligand was reacted with 1 equivalent of cobalt chloride hexahydrate using methanol as solvent and was stirred at 55 °C for 3 hours. After which, methanol was evaporated and the resulting solid was washed with cold diethyl ether to yield the chloride salts in quantitative yield. Bi-pyridyl (*tert*-butyl and methoxy) ligands were purchased from Sigma-Aldrich and used without purification. The preparation was performed following protocols in the literature.^37–39^

### MALDI-TOF synthesis product confirmation

Matrix-assisted laser desorption ionization time-of-flight mass spectrometry was performed for synthesis product confirmation. Each of the four complexes was dissolved in a 60% acetonitrile 0.1% trifluoroacetic acid (TFA) solution containing the carrier species sinapinic acid at a concentration of 20 mg mL^−1^. Samples were spotted onto an MSP96 target polished steel microSCOUT Target sample plate and ran on a Bruker Daltonics microflex MALDI-TOF mass spectrometer with a Bruker microSCOUT ion source. Spectra were normalized based on relative abundance before peak picking and analysis was performed.

### Solubility quantitation of compounds

Complexes 1–4 and 6 were initially dissolved in 60% acetonitrile and 40% ethylene carbonate w/v. Complex 5 was measured in 100% DI H_2_O. Samples were chemically oxidized with the addition of potassium ferricyanide to a concentration of 1.5 mM, with complex concentration at 300 μM. To determine the minimum concentration of organic solvent needed, the change in absorbance at the 295 nm peak in the oxidized spectra of complexes 1–4 was measured before and after centrifugation sedimentation for 2 min at 21 000×*g*. Large ΔAbs_295 nm_ values were taken to be indicative of the complex not being in solution, and ΔAbs_295 nm_ less than 0.05 a.u. as indicative of the complex being solubilized. Absorbance measurements were measured on the drop reader a NanoDrop One C. Measurements reported were taken in technical triplicate.

### UV-Vis spectra and redox difference spectra

For the UV-Vis oxidized and reduced spectra, redox difference spectra for complexes 1–4 and 6 were recorded in 33% acetonitrile, while the oxidized and reduced spectra were recorded in 50% acetonitrile. Complex 5 was measured in 100% DI H_2_O. The method for generating redox difference spectra is based on the initial chemical redox method published by Ke for PSI.^40^ Quartz split cuvettes were used to generate physically subtracted redox difference spectra while avoiding artifacts from either the oxidant or reductant species. The concentration of cobalt complex used was 0.2 mM, and 30 μL of either 25 mM potassium ferricyanide or sodium ascorbate was spiked in as the oxidant and reductant, respectively. Samples were brought up to 750 μL total volume in 50% acetonitrile. Baseline correction was performed using the MES buffer used in flash photolysis experiments and described below before sample spectra were taken. Spectra were taken using an Evolution 300 spectrophotometer after an hour incubation of samples in the dark at room temperature.

### Cyclic voltammetry/midpoint potential determination

Cyclic voltammetry was performed to determine midpoint potentials, stability after multiple redox events, and diffusional control analysis of these complexes. A glassy carbon electrode was used as the working electrode, a pseudo Ag/AgCl electrode was used as the reference (a true Ag/AgCl reference electrode was used for any aqueous measurements), and a platinum counter electrode was used. Measurements were taken using a Bio-Logic SP-50 potentiostat in acetonitrile and matching 0.1 M either tetrabutylammonium hexafluorophosphate (TBAPF), tetrabutylammonium perchlorate (TBAP), or NaCl supporting electrolyte, except for complex 5 which was measured in deionized H_2_O with NaCl supporting electrolyte. Each complex was added to a final concentration of 2.5 mM, and measurements were taken at scan rates of 10, 20, 50, 100, 150, 175, 200, and 250 mV s^−1^ for each cobalt complex. The software package QSOAS v2.2 was used for electrochemical data processing and analysis. For diffusional control analysis, the Randles–Sevcik equation^41^ was used to examine the effect of slew rate on peak currents. Cathodic peak heights were measured and plotted against both the scan rate and square root of the scan rate and linear regression on these plots was performed using GraphPad Prism 8.

### Organic solvent effects on PSI stability

PSI stability in various organic solvents was tested *via* 77 K chlorophyll fluorescence emission spectra. Solvents were made up using appropriate volumes of neat acetonitrile or ethylene carbonate and 50 mM MES buffer, pH 6.4 with 0.03% β-DDM and 25 mM MgSO_4_ for dilution to the desired concentration of organic solvent. PSI was added, samples were thoroughly mixed and incubated in the dark at room temperature (23 °C), and measured after both 1 and 24 hours. Samples were transferred into glass EPR tubes and were slowly frozen in liquid nitrogen. Chlorophyll fluorescence spectra were measured using a PTI Quantamaster Dual-channel fluorometer. The excitation light was set to 430 nm for excitation of chl *a* with a slit width of 0.75 nm and emission was scanned using 1 nm steps from 600–800 nm. Each spectrum was averaged from 3 traces. Baseline correction, peak maxima and areas were computed using Gaussian peak fitting in Origin Pro 2019.

### Spectral measurement of photo-oxidized P700^+^ PSI reduction kinetics

To assess the ability of the complexes to act as electron donors to PSI, single-pulse LED flash photolysis was performed using a JTS-100 spectrometer. The pulse sequence used was 20(D50 ms)G[150 000 μA]30 msHT200 μs{20 μs, 500, 2500 ms, D} for initial assessment of redox mediator donation to photo-oxidized PSI-P700^+^. PSI-P700^+^ was monitored at 705 nm and the actinic LED was “Actinic 1” which has a center wavelength of 630 nm. For titration of cobalt complex #2, the timescale was increased to 120 seconds. Measurements were performed using 30 nM P700 of PSI per sample in a ternary solvent system that was 60% 50 mM MES buffer containing 25 mM MgSO_4_ and 0.03% β-DDM at pH 6.4, 24% ethylene carbonate, and 16% acetonitrile, or fully in the MES buffer for aqueous experiments. Methyl viologen was added to a concentration of 1 mM as an electron acceptor, and sodium ascorbate was added to a concentration of 1 mM to act as a sacrificial electron donor. A 20 ms baseline was measured before the 630 nm actinic light was turned on to maximum intensity for 10 ms to photo-oxidize all the PSI present in the sample. Reduction of P700^+^ PSI was monitored using a second pulsed 705 nm wavelength probe beam. Twenty traces per sample were averaged to generate reduction curves. Monophasic exponential association curve fitting with 1000 iterations was used to model the Co #2 and DCPIP titration series and residuals plotting was also performed in GraphPad Prism 7. For comparison to the cobalt complexes in both organic solvent and aqueous solution, reduction of photo-oxidized P700^+^ PSI by a commonly used small molecule redox mediator 2,6-dichlorophenolindophenol (DCPIP) was performed.

### Biophotovoltaic device fabrication and measurement

Conductive FTO glass electrodes were doctor bladed with a TiO_2_ suspension and then sintered at 500 °C to generate FTO glass electrodes with a TiO_2_ nanoparticle semiconductor layer. Photoanodes were stored in Dri-Rite until use. PSI-utilizing devices had concentrated 3 mg mL^−1^ chl *a* PSI that was isolated as previously described above drop-casted onto the TiO_2_ and allowed to dry to the eye. Counter electrodes were generated by carbon deposition on the conductive side of a second FTO glass electrode. The 2 electrodes were then offset and mechanically compressed together, with dual clamps on opposing sides holding the device together, and electrolyte was introduced. Mediators were present at a concentration of 30 mM when included in the electrolyte. The fully aqueous electrolyte used was 40% 50 mM MES buffer, pH 6.4 with 0.03% β-DDM, 25 mM MgSO_4_, and 60% ethylene carbonate. The ternary electrolyte is the same as described for kinetic experiments above, with acetonitrile present at 16%. Devices were allowed to sit to allow for electrolyte integration for approximately 30 minutes before device testing and measurements. Device illumination was performed using a Schott KL-2500 light source with inset filter holder and Schott red light filter (MOS-258-303) for all red actinic light experiments. All photochronoamperometric measurements were taken using a Bio-Logic SP-50 potentiostat for data collection. Data plotting was done in origin Pro 2019 and Graphpad Prism 8.

## Results

### Cobalt redox mediator synthesis and characterization

A diagram of the synthesis scheme of all six complexes can be seen in Scheme 1. MALDI-TOF mass spectroscopy was performed for all six complexes to confirm product characterization.

### Spectroscopic characterization of cobalt redox mediators

**Fig. 2:**
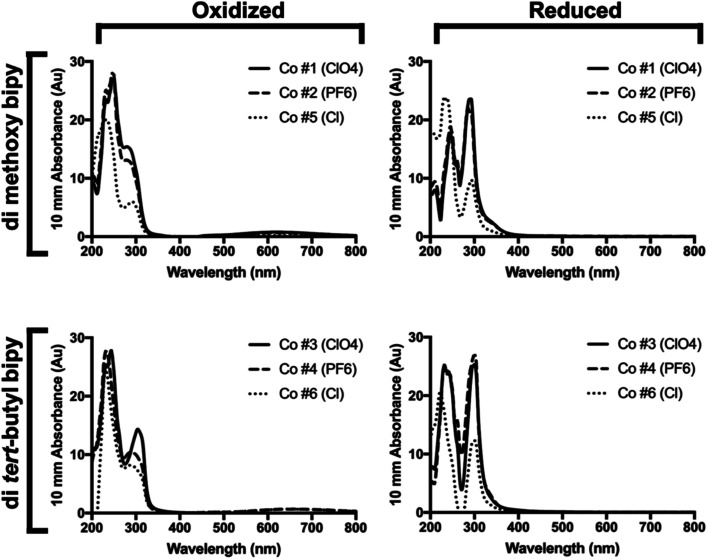
UV-Vis oxidized and reduced spectra of bipyridine Co redox mediator complexes. Shown are chemically oxidized and reduced UV-Vis spectra of all six di-methoxy and di-*tert*-butyl complexes at a concentration of 0.3 mM complex.

Chemically oxidized and reduced UV-Vis spectra taken in 50% acetonitrile for all but the bipy di-methoxy Cl complex (Co #5) can be seen in Fig. 2 and are similar to those previously reported for other cobalt complexes.^38,42^ This was necessary to confirm that these synthetic complexes would not compete with PSI for light absorption, which would reduce dye excitation efficiency in a biophotovoltaic device utilizing these complexes and PSI. There are two characteristic primary peaks present in the spectra at 250 and 295 nm. This indicates that the absorbance spectra of the redox mediator should not compete with the absorbance spectra of PSI. While both primary peaks are present in both the Co^2+^ and Co^3+^ oxidation states, the 250 nm peak dominates in the oxidized Co^3+^ state, while in the reduced Co^2+^ state the two primary peaks contribute approximately equally. When oxidized, all complexes also exhibited a low, broad absorption region from approximately 525–725 nm in addition to the two primary peaks. The absorbance spectra of both the oxidized and reduced forms of the complexes assayed do not have any significant overlaps with the absorbance spectrum of chlorophyll *a*, which is good from a device integration perspective, as these Co redox mediators should not be competing with PSI for dye photoexcitation energy.

**Fig. 3:**
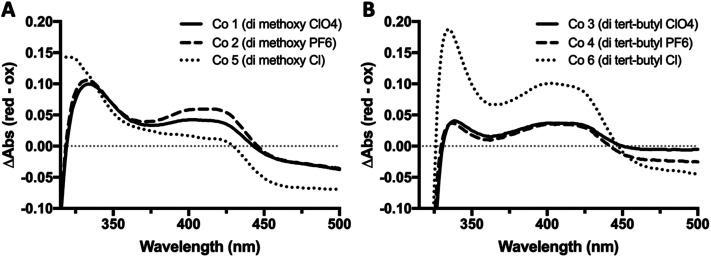
UV-Vis redox difference spectra of bipyridine Co redox mediator complexes. UV-Vis reduced minus oxidized difference spectra for all six complexes were obtained using quartz split cuvettes for physical spectral subtraction. Ferricyanide was used as a chemical oxidant, and ascorbate as reductant. Samples were prepared in a 1 : 3 acetonitrile : H_2_O and incubated in the dark for 1 h at 22 °C before taking spectra.

The reduced minus oxidized difference spectra were also taken for all six complexes and can be seen in Fig. 3, the di-methoxy complexes in Fig. 3A and the di-*tert*-butyl complexes in Fig. 3B. All six complexes have a peak in their redox difference spectra at 335 nm, and a low broad peak from 360–440 nm. These results are in line with previously reported spectra, where the 250 and 195 nm peaks that are seen in the sub-350 nm region were assigned based on the onset of the ligand-based π–π* transition associated with the electron transfer capabilities of these complexes.^38^

### Electrochemical characterization

**Fig. 4:**
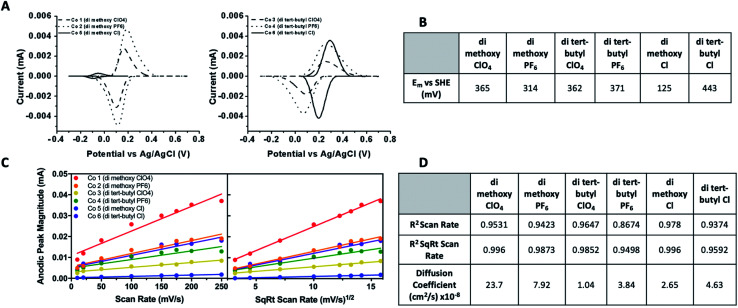
Electrochemical analysis of cobalt bipyridine redox mediator complexes. (A) Cyclic voltammograms with non-faradaic processes subtracted of cobalt complexes 1–6 *vs.* Ag/AgCl taken at a slew rate of 10 mV s^−1^. CVs were run on a glassy carbon electrode at 8 slew rates varying from 10–250 mV s^−1^. Complexes 1–4 and 6 were measured in acetonitrile, 5 in aqueous solution. (B) Midpoint potentials *vs.* SHE for all complexes. (C) Plots of anodic peak current *vs.* both scan rate and square root of scan rate. (D) Goodness of fit of linear regression of plots in 5C given as *R*^2^ values, along with calculated diffusion coefficients.

Cyclic voltammetry was performed to determine midpoint potentials, assess the reversibility, and calculate diffusion coefficients of these synthetic Co complex redox mediators. The cyclic voltammograms, diffusion control analysis plots, and calculated midpoint potentials (*E*_m_) and diffusion coefficients are given in Fig. 4. The midpoint potential of *T. elongatus* PSI has been measured as 423 mV *vs.* SHE, though PSI from various species has been shown to exhibit a large window (∼80 mV) of species dependence.^43,44^ Cytochrome *c*_6_ from *T. elongatus*, the native *in vivo* electron donor to PSI, has an *E*_m_ of 329 millivolts (mV) *vs.* SHE, which is in line with the 320–350 mV *vs.* SHE reported values for cytochrome *c*_6_ from other cyanobacterial and algal species.^45^ The *E*_m_ for all six cobalt redox mediator complexes were between 125–443 mV *vs.* SHE. Most are similar or slightly more negative than cytochrome *c*_6_, the *in vivo* metalloprotein redox mediator for PSI in *T. elongatus*. Interestingly, the di-*tert*-butyl bipyridine complex with a Cl counterion is considerably more positive by approximately 100 mV, however the chemical basis of this shift is not clear. This suggests that at least 5 of the 6 complexes assayed here should have sufficient driving potentials for electron donation to PSI. The measured *E*_m_ for complexes 1–6 were, in order, 365, 314, 362, 371, 125, and 443 mV *vs.* SHE.

Cyclic voltammetry was performed as a function of scan rate to reveal details about reversibility, thermodynamic potential for electron donation to PSI, and the interactions between the cobalt redox mediators and electroactive surfaces. A linear relationship between peak height and the square root of the scan rate (Fig. 4D) indicate reversible diffusion controlled processes according to the Randles–Sevcik equation,^41^ as well as the electrochemical species being studied not adsorbing to the electrode surface. This is a necessary characteristic for a redox mediator to be used in a biophotovoltaic device, to keep photosensitive PSI dye free and available for further photoexcitation events. Diffusion coefficients were also calculated using the Randles–Sevcik equation^41^ and are given in the table in Fig. 4D.

Next, the minimal concentration of acetonitrile needed for complex solubilization had to be determined in order to minimize the amount of organic solvent needed to keep PSI in as much of an aqueous environment as possible. To this effect, solubility of the cobalt complexes in increasing concentrations of acetonitrile was measured. Acetonitrile was specifically focused on instead of ethylene carbonate as acetonitrile has previously been identified as being key for redox mediator solubility while ethylene carbonate is commonly used to increase the ionic conductivity of electrolytes due to its high dielectric constant. To assess this, the change in the amplitude of the 295 nm peak in the reduced spectra was monitored by measuring pre- and post-centrifugation as a solubility indicator. A larger ΔAbs value was understood to mean that less of the complex was in solution, and a smaller ΔAbs value that the complex was well-solubilized.

**Fig. 5:**
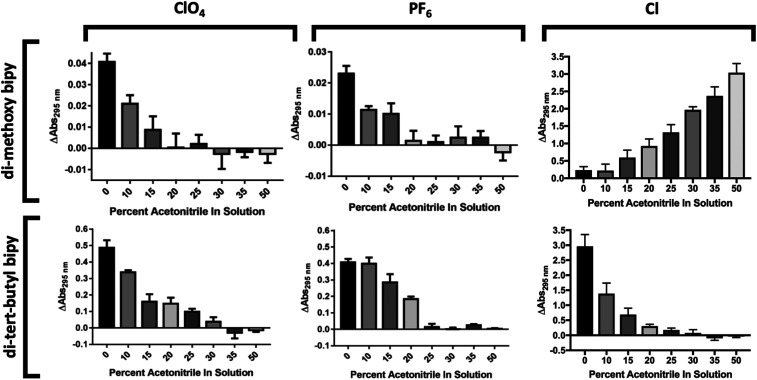
Solubility of cobalt redox mediators in acetonitrile : H_2_O binary solvent systems. The solubility of all six complexes in increasing percentages of acetonitrile was assessed to determine minimum concentration of acetonitrile needed for solubility. All samples were chemically oxidized prior to measurement. Error bars represent SEM.

Results are shown in Fig. 4. Any insoluble material should contribute to absorbance pre-centrifugation but not post-centrifugation. The results of the acetonitrile solubility analysis can be seen in Fig. 5. The di-methoxy complexes with PF_6_ and ClO_4_ counterions were determined to be soluble in water–acetonitrile mixed solvents with at least 20% v/v acetonitrile while the di-*tert*-butyl complexes with PF_6_ and ClO_4_ counterions were determined to be soluble in a mixed aqueous solvent with at least 25% v/v acetonitrile. The di-methoxy complex with Cl as counterion proved to be soluble in water, starting to crash out at concentrations of approximately 20% and the di-*tert*-butyl complex with Cl as counterion proved to be soluble in concentrations of acetonitrile exceeding approximately 20%.

### PSI structural stability in organic solvent

A significant challenge for utilizing synthetic redox mediators with a biological dye such as PSI is their low solubility in aqueous solutions that are native for PSI. Initially, it was necessary to identify a solvent system that would allow for further characterization of these cobalt redox mediator complexes. The ratio of organic solvent used was systematically varied to optimize the simultaneous solubility of PSI and the cobalt mediators. In addition to the solubility of PSI, stability was another concern since organic solvents can extract chlorophyll and lipophilic cofactors from PSI, potentially altering light-harvesting and photochemistry capabilities. While numerous studies have been performed on pigment extraction, we are not aware of any studies to determine the upper limit of solvents that will not significantly alter the cofactor composition of PSI.^46–48^

**Fig. 6:**
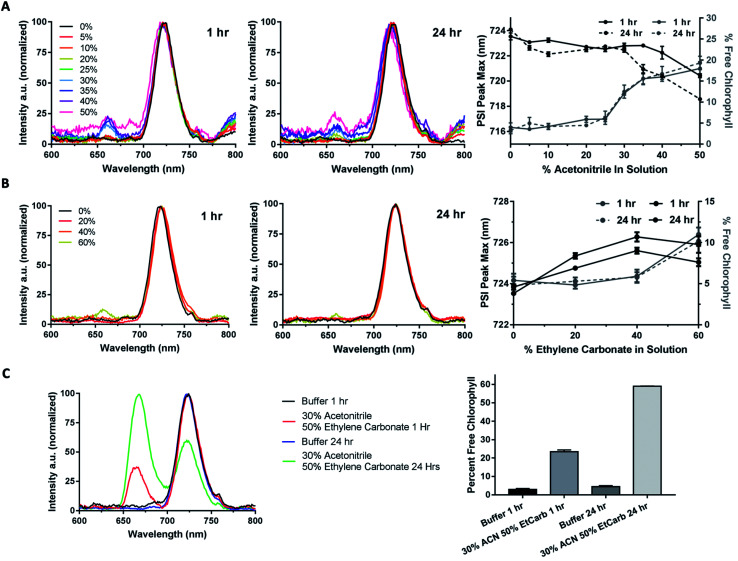
Organic solvent effects on PSI structural stability. Organic solvent effects on PSI stability were assessed using 77 K low temperature chlorophyll fluorescence emission spectra. Baseline correction was performed, peak intensities of the PSI peak were normalized, and percent free chlorophyll (660 nm) along with PSI-bound chlorophyll (720 nm) shift were calculated at 1 and 24 hour timepoints from Gaussian curve fitting. All spectra shown are the average of triplicate results. (A) 77 K chlorophyll fluorescence emission spectra of 1 and 24 hour timepoint of PSI in 0–50% acetonitrile. Left panel 1 h timepoint, center 24 h timepoint, right panel PSI peak shift and chlorophyll loss quantification. (B) 77 K chlorophyll fluorescence emission spectra of 1 hour and 24 hour timepoint of PSI in 0–60% ethylene carbonate. Left panel 1 h timepoint, center 24 h timepoint, right panel PSI peak shift and chlorophyll loss quantification. (C) 77 K chlorophyll fluorescence emission spectra of 1 hour and 24 hour timepoint of PSI in 30% acetonitrile + 50% ethylene carbonate, 20% aqueous buffer. Right panel is chlorophyll loss quantitation.

Although a synthetic Co complex has been incorporated once before in a PSI-based biophotovoltaic device as the redox mediator, the solvents used for that complex may not have been optimal for sustained stability and activity of PSI.^30^ The stability of PSI was tested using low temperature fluorescence spectroscopy in the organic solvents required by the majority of the cobalt complexes for solubility and electrochemical activity. Low temperature fluorescence spectroscopy reveals the local environment of non-covalently attached chlorophyll molecules in both PSII and PSI to be evaluated.^49^ To assess the structural stability of PSI in various concentrations of organic solvents, 77 K low-temperature fluorescence spectra were taken after both 1 or 24 hour incubations in varying concentrations of acetonitrile and ethylene carbonate, as seen in Fig. 6. At 77 K, upon excitation in the chlorophyll *a* Soret band (420–450 nm), intact cyanobacterial PSI has a characteristic fluorescence emission peak at approximately 720 nm. Free chlorophyll that is not bound in a reaction center like PSI or a light harvesting antenna protein has a characteristic emission peak at approximately 660 nm upon similar excitation in the Soret band. The presence of a 660 peak in a fluorescence spectrum of purified PSI indicates decomposition and loss of pigments and other lipophilic cofactors.

In up to 25% v/v acetonitrile in solution, no measurable chlorophyll was extracted from PSI over the 24 hour treatment (Fig. 6A, left and center panels). However, as the acetonitrile concentration was increased up to 30–40% solvent in solution, approximately 20% of the chlorophyll in the PSI was extracted. This extraction seemed to reach completion, as measurements of the same sample after 24 hours revealed no further increase in free chlorophyll was seen, shown in the right panel of Fig. 6A. Together these results suggest the presence of a labile pool of chlorophyll that can be removed from PSI using acetonitrile with minimal structural effects to the overall protein–pigment complex. Peak position of the 720 nm PSI peak was also measured, and a blue-shift of nearly 2 nm after 1 hour and approximately 5 nm after 24 hours was noted, and quantification is shown in Fig. 6A, right panel. This blue-shift suggests that in increasing amounts of acetonitrile the local environment of the far-red chlorophylls associated with PSI^50–52^ may be altered by possibly removing either bound lipids or detergent molecules from the complex beyond leaching out chlorophyll.

Similarly, the effects of ethylene carbonate on PSI structural stability were performed. Ethylene carbonate is an organic solvent commonly used in the liquid heterojunctions of DSSCs, utilized to promote electrolyte conductivity due to its high dielectric constant. PSI remained stable in ethylene carbonate solutions up to 60% w/v over 24 hours, seen in Fig. 6B left and center panels. Both 20 and 40% ethylene carbonate in solution led to no more chlorophyll loss from PSI than aqueous MES buffer with 0.03% DDM for detergent exchange. When 60% ethylene carbonate in solution was reached, only approximately 10% of total chlorophyll was lost from PSI, seen in Fig. 6B right panel. Upon performing peak fitting to assess PSI 720 nm peak position shift, there was a moderate red-shift of approximately 2 nm that was stable up to 24 hours in solution.

Interestingly, when assessing the structural stability of PSI in a ternary solvent system of 30% acetonitrile, 50% ethylene carbonate, and 20% aqueous MES buffer containing 0.03% β-DDM, the two organic solvents had a synergistic destructive effect on PSI, shown in Fig. 6C. This ternary system was of interest as a 40% ethylene carbonate and 60% acetonitrile binary solvent system has been used previously for integration of Co-based redox mediators in a PSI biophotovoltaic device.^30^ While neither organic solvent was disruptive to the chlorophyll bound to PSI on their own, our results show that they have a much more pronounced effect on the structural destabilization of the PSI complex together in solution. However, in the previous study by Mershin *et al.*, PSI was already bound to a semiconductor electrode, and the effect of the 40% ethylene carbonate, 60% acetonitrile solvent system in that context on PSI stability over time remains unknown. After 1 hour of incubation in this solvent system, nearly 25% of chlorophyll had been leached out of the PSI, and after 24 hours over 60% of the chlorophyll had been pulled out of the bound light harvesting antenna chlorophyll, shown in the right panel of Fig. 6C. So much chlorophyll loss suggested severe destabilization of the PSI structure and also that this ternary system at such a high concentration of organic solvent would not prove useful in future biophotovoltaics research for integration of PSI into devices. For kinetics experiments performed next, it was determined based on the binary solvent system titrations in Fig. 6 that a ternary solvent system of 60% aqueous MES buffer, 24% ethylene carbonate, and 16% acetonitrile would be sufficient to minimally affect PSI stability while still retaining enough of the organic solvent to keep the cobalt redox mediators in solution.

### Cobalt complexes donate electrons directly to PSI

Using the same solvent system that was both compatible with PSI and able to solubilize the cobalt redox mediators over a period of at least 24 hours, the ability of cobalt complexes to donate electrons to PSI directly was investigated. Although cobalt-based redox mediators have been used once previously in a PSI-based biophotovoltaic device by Mershin *et al.*,^30^ that work did not test for either complex solubility, PSI stability, or verify that the cobalt redox mediators in the device were in fact directly reducing PSI. To track PSI reduction kinetics, flash photolysis using a Joliot-type spectrophotometer (JTS-100, Bio-Logic, Grenoble). In a flash photolysis experiment to track PSI reduction kinetics, a 630 nm actinic pulse(s) is used to photo-oxidize all of the PSI present in the sample, and then a second beam (either 705 or 810 nm) is used to measure and track the reduction of P700^+^ as a function of time. We have developed this assay for the previous generation JTS-10 instrument and described it in detail in previous work.^53^

Kinetics were tested over 2.5 seconds at first to initially assess ability to donate to PSI. The resulting kinetic traces were fit with a monophasic exponential association equation, and the resulting observed rates are shown in Table 2. A general equation of monophasic exponential association is shown in eqn (1), where *Y* is the value of *Y* when *X* = 0, *Y*_max_ is the amplitude of the phase, *k* is the observed rate, and *t* is time. As shown in the kinetics fitting results in Table 2, when tested in the 60% aqueous, 24% ethylene carbonate and 16% acetonitrile ternary solvent system showed that the first four complexes (di-methoxy ClO_4_ and PF_6_, di-*tert*-butyl ClO_4_ and PF_6_) do in fact donate electrons to PSI *in vitro*.

**Table 2:** Cobalt redox mediators donate directly to PSI *in vitro*. The initial results assessing the ability of these cobalt complexes to directly donate to photo-oxidized P700^+^ PSI was assessed over a timescale of 2.5 s. When no donor was present, no recovery of photo-oxidation was seen and a long-lived P700^+^ PSI signal was seen. All complexes were present at 10 mM concentration

	Di-methoxy ClO_4_ Co#1	Di-methoxy PF_6_ Co#2	Di-*tert*-butyl ClO_4_ Co#3	Di-*tert*-butyl PF_6_ Co#4
*k*_obs_ (s^−1^)	0.014	0.028	4.0 × 10^−5^	4.6 × 10^−5^

Monophasic exponential association general equation:

1 *Y* = *Y*_max_ × (1 − *e*^−*kt*^)

**Fig. 7:**
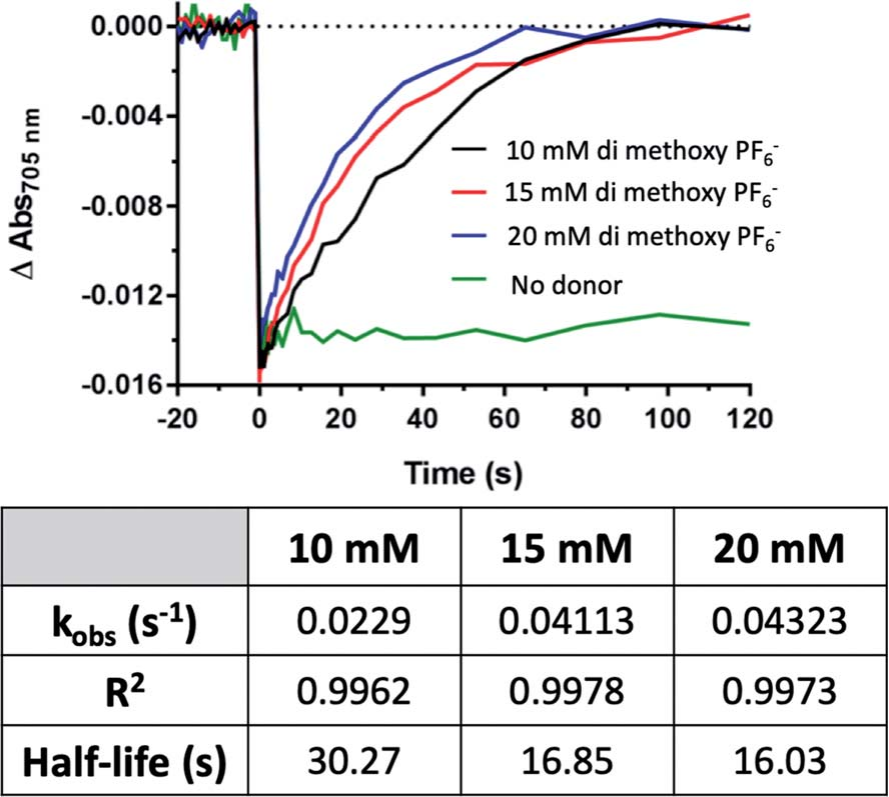
Rate of PSI-P700^+^ reduction increases linearly with redox mediator concentration. Above concentrations of 10 mM, rates of electron transfer increased linearly with the addition of Co redox mediator #2, the di-methoxy PF_6_^−^ variant. Kinetics traces were fit to single-phase exponential association equations, the observed rates, *R*^2^ and calculated half-lives of which can be seen in the table.

The di-methoxy PF_6_ Co#2 variant of these bipyridine complexes was selected for further kinetics studies as it had the greatest initial observed rate (*k*_obs_). Kinetics were measured at concentrations of donor (Co #2) at 10, 15, and 20 mM, and monitored over 120 s until return to baseline was achieved (Fig. 7). A linear increase in the rate of electron transfer to PSI-P700^+^ was observed when rates were plotted against donor concentration, plotted in Fig. 10. At concentrations above 20 mM, the Co complexes crashed out of solution and multiple attempts at keeping the high concentration of redox mediator in solution did not prove successful, even with the ternary solvent system that was being used to try and balance the needs of both PSI and these Co redox mediators.

**Fig. 8:**
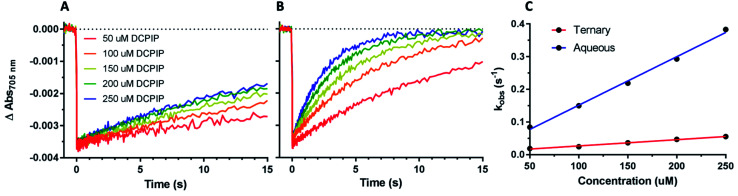
Effect of organic solvent on PSI-P700^+^ reduction kinetics By DCPIP. (A) DCPIP titrations were performed in the ternary 60% aqueous, 24% ethylene carbonate, and 16% acetonitrile solvent system. Concentrations are increasing from 50 μM (red)–250 μM (blue). (B) DCPIP titrations were performed in fully aqueous MES buffer. (C) Observed rates of PSI-P700^+^ reduction plotted against concentration of DCPIP.

**Fig. 9:**
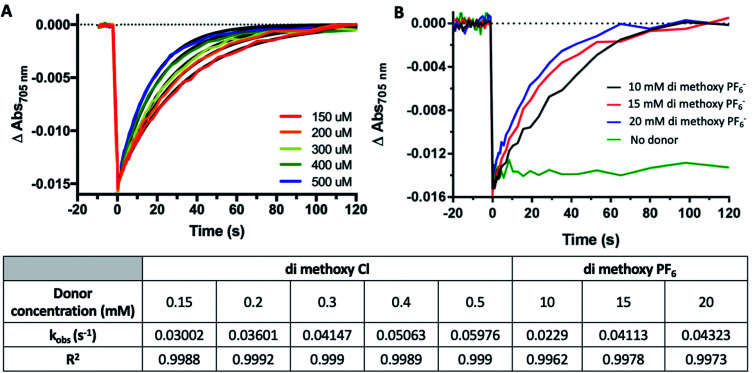
Organic Solvent Slows PSI-P700^+^ Reduction Kinetics by Co complexes. (A) Co #5 di-methoxy Cl^−^ PSI-P700^+^ reduction kinetics from 150 μM (red) to 500 μM (blue). (B) Co #2 di-methoxy PF_6_^−^ PSI-P700^+^ reduction kinetics from 10–20 mM. Observed first order rate constants (*k*_obs_) are given in the table below for each trace along with *R*^2^ of monophasic exponential fitting.

**Fig. 10:**
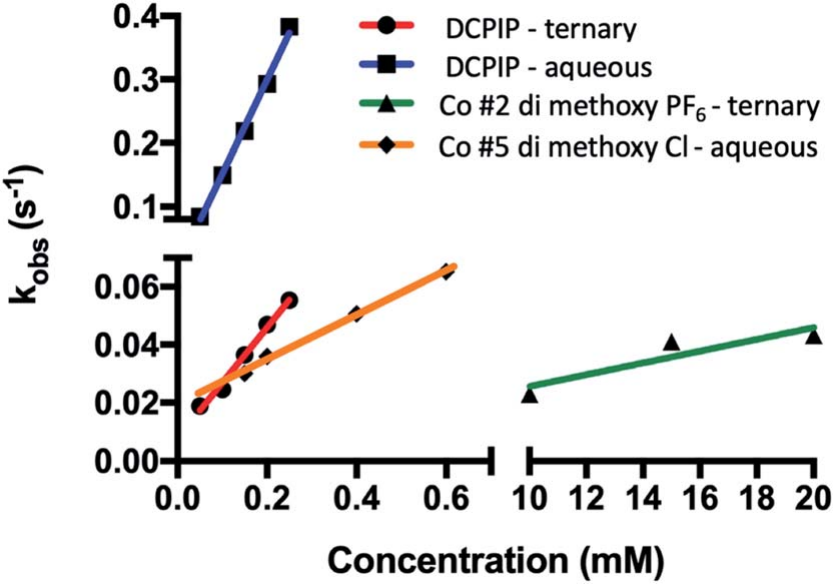
Organic solvents slow rates of PSI-P700^+^ reduction by redox mediators. Observed rates were plotted against concentration of redox mediator used in each trace. Linear regression was then performed, and bimolecular rate constants were taken from the slopes of the linear regression.

### Effect of organic solvent on PSI reduction rates

While the synthetic cobalt-based complexes acted as redox mediators to reduce PSI in a dose-dependent manner, the effect of organic solvents on the reduction remained to be determined. Buffer composition has been shown to have effects on PSI-P700^+^ reduction kinetics even in aqueous solution.^53^ To initially assess the effects of organic solvent on PSI-P700^+^ reduction kinetics, a commonly used synthetic small molecule PSI electron donor, 2,6-dichlorophenolindophenol (DCPIP)^54–58^ that is soluble in both aqueous and organic solvent systems was utilized for direct comparison in both strictly aqueous and ternary buffer systems. In the ternary solvent system with a 40% organic solvent component (24% ethylene carbonate, 16% acetonitrile, 60% aqueous buffer) utilizing DCPIP concentrations of 50–250 μM, similar rates were observed as approximately 100-fold higher concentration of the di-methoxy PF_6_^−^ Co complex #2 was used, as seen in Fig. 8A. PSI-P700^+^ reduction kinetics of an identical titration series of DCPIP in fully aqueous buffer is shown in Fig. 8B. Next, observed rates were plotted against redox mediator concentration to generate a rate plot, and observed bimolecular rate constants were taken from the slopes of the rate plot in Fig. 10. Shifting from a ternary to a fully aqueous solvent system yielded an ∼7.9 fold increase in the observed bimolecular rate constant: 0.19 s^−1^ L mmol^−1^ for reduction in the ternary solvent system as compared to 1.50 s^−1^ L mmol^−1^ in the fully aqueous MES buffer.

Our results on the effects of the ternary buffer on the stability of the chlorophyll pigments in PSI and on PSI-P700^+^ reduction kinetics by DCPIP led us to assess the PSI-P700^+^ reduction kinetics of Co #5 – the di-methoxy Cl^−^ variant. This complex was fully soluble in water as indicated by the spectra and lack of precipitate. PSI-P700^+^ reduction kinetics by this complex in fully aqueous solution were measured at donor concentrations of 10, 15, and 20 mM. A comparison of the data in Fig. 7 of the organic solvent-soluble Co #2 – di-methoxy PF_6_^−^ to the kinetics was performed. The traces for these experiments can be seen in Fig. 9 below, along with donor concentration used, observed rates, and the goodness of the monophasic exponential fitting performed. However, by comparison of the two complexes PSI-P700^+^ reduction kinetics, the bimolecular rate constant of PSI-P700^+^ reduction is approximately 38 fold greater for the di-methoxy Cl^−^ Co#5 complex than the PF_6_^−^ counteranion Co#5 complex. All observed rates were plotted against concentration of donor used to generate a comprehensive rate plot for DCPIP and the di-methoxy complexes tested in both the aqueous and ternary solvent systems, shown in Fig. 10.

### Photocurrent generation by Co redox mediator biophotovoltaic devices

**Fig. 11:**
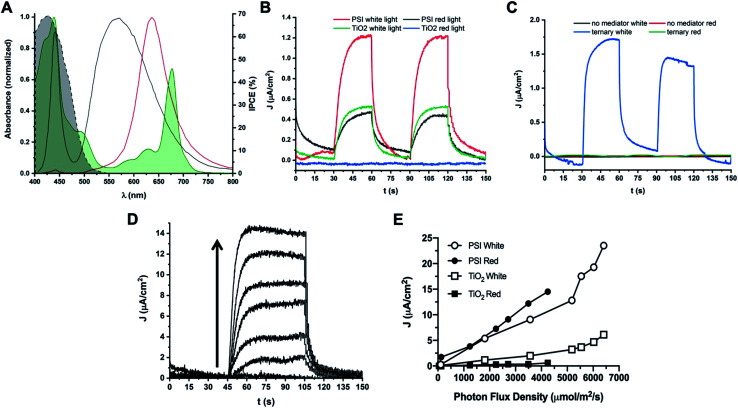
Photocurrent densities obtained from PSI-sensitized biophotovoltaic devices. Photocurrent densities of PSI-sensitized devices are reported. (A) Spectral irradiance of white and red illumination sources used. White light used for photocurrent experiments is shown in black, red light is shown in red. The UV-Vis absorbance spectrum of PSI is shown overlaid in green and is partially filled in. The dashed black line represents the percent Incident Photon-to-electron Conversion Efficiency (IPCE) of TiO_2_ semiconductor. (B) Photocurrent densities of cells with Co di-methoxy Cl^−^ mediator #5 in fully aqueous electrolyte with and without PSI as sensitizer. Photocurrent densities in both white and red (PSI-specific) light are reported. Light was at 10% capacity of illumination source for all traces (1800 μmol m^−2^ s^−1^ for white light, 125 μmol m^−2^ s^−1^ for red light). (C) Photocurrent density generation with no mediator present in electrolyte, or the Co di-methoxy PF_6_^−^ mediator # 2 in the ternary buffer mixture (blue and green) described in previous experiments. (D) Photocurrent density of PSI-Co di-methoxy Cl mediator #5 device as a function of red light intensity measured immediately after device fabrication. Arrow shows direction of increasing light intensity for each trace. Data is quantified in (E). (E) Photocurrent density plotted against PFD of light used for illumination. Data for cells with the aqueous Co di-methoxy Cl^−^ mediator #5 and with or without PSI present under both red and white light illumination are shown.

Both direct electron donation to PSI by our synthetic Co mediators and stability of PSI in organic solvent have been demonstrated *in vitro*; subsequently, biophotovoltaic devices were assembled and tested. We utilized a white light illumination source capable of photoexcitation of both PSI and TiO_2_ semiconductor, and a Schott red light filter (MOS-258-303) to generate PSI-specific actinic light. The spectral irradiance of both light sources overlapped with the PSI absorption spectrum at RT is shown in Fig. 11A to show the specificity of the red actinic illumination.

Devices with the aqueous mediator, complex Co#5 present in the electrolyte but no PSI present as the photosensitive dye showed photocurrent generation under white light which is capable of TiO_2_ photoexcitation, but no photocurrent in the same device was observed when using PSI-specific red light for device illumination, shown in Fig. 11B. When PSI is present as the dye with the aqueous mediator Co#5 in the electrolyte, increased photocurrent density is measured under white light illumination, but there is still 0.5 μA cm^−2^ generated under low levels of red light illumination for the same cell. When PSI was present, and mediator was absent in the electrolyte, no photocurrent was generated under either white or red light illumination, shown in Fig. 11C, as expected if the device circuit is not completed. When the Co#2 mediator in the ternary electrolyte buffer described previously was used along with PSI as the photosensitive dye in a device, photocurrent was only observed under white light illumination, as shown in Fig. 11C. This suggests that either interaction of PSI with the TiO_2_ semiconductor was interrupted, or the photoactivity of PSI was lost due to use of the ternary electrolyte containing organic solvents. Generally, a slow rise and fall in the current is seen in the photocurrent data reported, which may be due to the need for optimized electrode interaction for both the photoanode and the cathode where the mediator accepts electrons. Previous work has shown the use of PEDOT cathodes can help to improve the output of Co redox mediator – based devices and that electrode interaction optimization is key to performance improvement.^59^

Photocurrent density as a function of light dependence of a biophotovoltaic device with PSI and the aqueous Co#5 mediator in the electrolyte was also measured, shown in Fig. 11D. Photocurrent densities increased linearly with respect to increasing photon flux density, generating up to 14.5 μA cm^−2^ under nearly 4500 μmol m^−2^ s^−1^ red photon flux density (PFD), and up to nearly 25 μA cm^−2^ under white light illumination. The exact values and light intensities are given in Fig. 11E. TiO_2_ only devices with no PSI present as the photosensitive dye were similarly measured, and essentially no photocurrent was seen under red light illumination, and white light illumination did not yield as much photocurrent at all light intensities measured as compared to PSI-containing devices. Also seen in Fig. 11E is a steeper increase in photocurrent densities in devices at the highest light PFDs tested, a trend that is not seen in the PSI-specific red-light illumination values. This may be due to increased current coming from the semiconductor at high light intensities as the same trend is seen in devices with and without PSI.

Overall, this increase in PSI-specific photocurrent density for our aqueous mediator along with comparison to the loss of PSI-specific photocurrent and previously shown effects to PSI stability upon exposure to organic solvents suggests that development of bio-compatible and aqueous-soluble redox mediators is key for further improvw2n the future.

Taken together, this work suggests that previous work done on incorporating PSI into DSSCs that utilized organic solvents in their electrolytes may very well have decreased rates of PSI reduction as well as PSI and redox mediator stability. A much greater increase in the bimolecular rate constant was noted for the di-methoxy complexes than for DCPIP when moving from a ternary organic solvent system to a fully aqueous system, and in a biophotovoltaic device, no photocurrent was measured when using the Co #2 redox mediator that required organic solvent in the electrolyte. Furthermore, the ease of synthesis of these cobalt complexes and the ability to relatively easily adjust the functional groups and surface chemistry surrounding the active Co center as compared to traditional small molecule PSI donors such as DCPIP points to these cobalt complexes being a suitable system for further development and research to improve interactions with both the photoactive dye and the counter electrode. For example, these include potentially synthesizing scaffolds that these complexes can be inserted into in addition to the ability to modulate midpoint potentials through the electron-withdrawing/donating nature of functional groups as well as ligand backbone structure. These results point to the development of abundant transition metal based redox mediators as a continued area of interest for optimization in biophotovoltaics development.

## Discussion

An emerging technology in solar energy based on sustainable low-cost materials, relatively simple manufacturing processes, and high functionizability is dye-sensitized solar cells (DSSCs). An increasing number of DSSCs are in fact biophotovoltaic devices,^5,6^ utilizing PSI due to the high abundance of material, the near unity internal quantum efficiency of the PSI reaction center, and the low environmental impact of material production. However, reduction rates remain low for the cyanobacterial PSI commonly used in these devices and are an impedance to increasing photocurrent densities and efficiencies of these devices.

The most commonly used DSSC redox mediator for photosensitizer reduction, the iodide/triiodide (I^−^/I_3_^−^) couple, is incompatible with biophotovoltaics as it is corrosive to most metal as well as protein (potentially from UV-generated I_2_^−^ and I^−^ radical species),^60^ has a redox potential close to PSI giving low driving potentials, and has significant absorption in the visible region of the electromagnetic spectrum, reducing the energy available for photoexcitation of the PSI photosensitizer. As such an area of interest has been the incorporation of synthetic redox mediators using other transition metals as redox-active centers, such as cobalt. Previous work has identified cobalt complexes to try and replace the canonical iodide/triiodide pair, with focus on polypyridine-based coordinating ligands.^61,62^ Nearly all cobalt-based synthetic redox mediators published to date have required organic solvents for solubility, utilizing a 60 : 40 ethylene carbonate/acetonitrile solvent system.^30,38,42,63^ However, studies have not attempted to reconcile the incompatible solubility and activity requirements for both synthetic redox mediators and PSI in a manner that also promotes greater IPCEs and photocurrent densities.

The work presented here focuses on the synthesis and characterization of cobalt-based redox mediator complexes and their ability to donate electrons to PSI. We have explored the effect of organic solvents and optimized a solvent system that balances the solubility and stability requirements of both the dye and PSI complex for biophotovoltaic device integration. The use of organic solvents in the liquid heterojunction of DSSCs is unfavorable for a number of reasons, including the high volatility, toxicity, and/or explosive nature of many of these solvents^64^ along with leaking of water into the device either during manufacturing or during usage. A goal of DSSCs in terms of predicted performance is thin, flexible, aqueous-based devices.^64–67^ Though some work has been done on adjusting the properties of cobalt-based polypyridyl complexes to make them compatible with aqueous electrolytes, this remains an area of research where more work is needed.^67^

There is a need to further investigate the effects of organic solvents on PSI stability and electron transfer kinetics for biophotovoltaics, beyond just the recent interest in the field in moving away from organic solvents in liquid heterojunctions as electrolyte carriers for all DSSCs, not just biologically-based ones.^64^ Of particular interest are two reports,^34,36^ where a nearly 20-fold increase in current density was obtained when moving from a 50% diethyl ether to a fully aqueous liquid heterojunction. One of the highest reported photocurrent densities utilizing PSI as the dye in a DSSC have used cobalt-based synthetic redox mediators that require organic solvent electrolytes. However, the effects these electrolytes have on PSI were not assayed.^30^ Here, our results showed that the electrolyte used in PSI-based biophotovoltaics plays a significant role in PSI dye stability and activity as well as PSI reduction rates.

Key solubility conditions were investigated which enabled both PSI and synthetic redox mediators to be stabilized in solution in tandem. It was found that a 60% aqueous, 24% ethylene carbonate and 16% acetonitrile solution allowed for both PSI and PF_6_^−^ or ClO_4_^−^ counteranion-based complexes to be soluble and retain enough activity to function together constructively for kinetics assays and for future biophotovoltaic integration. In binary solvent systems of acetonitrile up to 50%, a labile pool of up to 20% total chlorophyll was leached out of the protein–pigment complex. This imparts a twofold negative effect: the chlorophyll molecules not bound within the PSI complex reduce light harvesting capabilities of the complex, and free chlorophyll in solution competes for light with PSI, leading to more light that is not efficiently funneled into performing charge separation. However, in ethylene carbonate binary systems up to 60%, there was an approximate increase in free chlorophyll of only 5% over 24 hours. There are 201 water molecules in the crystal structure of *T. elongatus* PSI^13^ involved with coordination of antenna chlorophyll and protein subunits, of which the majority are coordinated with core protein PsaA and PsaB that also bind the internal cofactors used for electron transfer through the complex. Organic solvents may be disrupting this network of water-based hydrogen bonding, leading to the leaching of chlorophyll and slowed PSI reduction rates reported here.

Herein, we have demonstrated that synthetic cobalt complexes are able to directly donate electrons to PSI, as no PSI reduction was seen in the absence of donor over 120 s, and is consistent with previous work using PSI *in vitro* for energy conversion. In the previous work, no electron donation to PSI was observed when 1 mM ascorbate and no cytochrome *c*_6_ was present in a hydrogen producing cell.^10^ The electron transfer kinetics between PSI and Co#5, the tris(4,4′-di-methoxy-2,2′-bipyridine)cobalt(ii) chloride mediator are faster than the other cobalt complexes studied here, presumably due to solvation, structural and counteranion properties. We have shown that this occurs in a linear dependence on concentration of donor when measuring PSI-P700^+^ reduction kinetics in solution, further supporting the claim of direct electron transfer from these cobalt complexes to PSI. Experiments testing effects on mediator concentration on photocurrent generation in biophotovoltaic devices are the subject of future work. Moving to a fully aqueous system increased PSI reduction rates approximately 8 fold for DCPIP, and approximately 38 fold for the di-methoxy Cl^−^ Co#5 variant of the cobalt complexes presented here.

We also report differential photocurrent generations for PSI- and Co-based redox mediator biophotovoltaic devices, with photocurrent under PSI-specific red light illumination seen for the aqueous di-methoxy Cl^−^ mediator Co#5, and no photocurrent generated with the di-methoxy PF_6_^−^ mediator Co#2 present in the ternary electrolyte described. This may be due to either interruption of PSI's photoactivity or its ability to interact with the TiO_2_ semiconductor, though further studies are needed to assess this. Increased photocurrents up to 25 μA cm^−2^ were seen in PSI – Co di-methoxy bipy Cl^−^ Co#5 devices. Also of note is an early study on Co-based DSSC redox mediators, where the authors found that bulkier counteranions yielded lower photocurrent densities in devices.^42,68^ Further work by Masconi *et al.* on Co redox mediators found that these mediators often form ion pairs with the very common ruthenium-based dyes used in DSSCs, leading to high levels of charge recombination and further decrease in photocurrents, which further strengthened our interest in pairing Co redox mediators with a biological dye like PSI.^69^ Future work will aim to help improve the electrode interfaces of these devices now that more information is known on dye/electrolyte interactions in our PSI-based biohybrid devices, as previous work on fully inorganic systems found significant improvements when modifying the cathode material.^59^

To date, this is the first report on the stability and reduction kinetics of PSI by synthetic cobalt-based redox mediators in non-organic solvents with relevance for biophotovoltaic applications, many of which are commonly used to enhance exciton and hole generation in devices such as solar cells. This is also, to our knowledge, the first report focused on the design of Co redox mediators as PSI electron donors that allows for optimization in multiple areas including solvent compatibility, redox midpoint matching, reduction kinetics, avoidance of spectral overlap, cost and stability of both the redox mediator and PSI. Further efforts for fine-tuning both solubility and increased affinity for PSI for synthetic redox mediators *via* introducing new functional groups and counteranions is clearly needed and ongoing, along with cathodic modifications in devices. Overall, this work suggests that one of the primary limitations for PSI-based biophotovoltaic devices utilizing synthetic redox mediators is solubility and incompatible solvent systems rather than any innate incompatibility between the synthetic redox mediators and PSI, and that bio-inorganic devices may yield improvements in device stability over time. Further research and improvements on PSI-based biophotovoltaics may yield more feasible bio-hybrid devices in the future with more competitive efficiencies and production costs.

## Conflicts of interest

There are no conflicts of interest to declare for the authors.

## Supplementary Material
